# Chemokines and phosphatidylserine: New binding partners for apoptotic cell clearance

**DOI:** 10.3389/fcell.2022.943590

**Published:** 2022-08-25

**Authors:** Sergio M. Pontejo, Philip M. Murphy

**Affiliations:** Laboratory of Molecular Immunology, National Institute of Allergy and Infectious Diseases (NIH), Bethesda, MD, United States

**Keywords:** efferocytosis, apoptotic bodies, extracellular vesicles, cell migration, phagocytes, phagocytosis, find-me signals, breadcrumb

## 1 Introduction

Efferocytosis refers to efficient, non-inflammatory and immunologically silent elimination of dead and dying cells by phagocytes and is an essential and general process for maintaining homeostasis. Efferocytosis mechanisms are triggered by two main classes of signals generated by the dying cell itself: “find-me” signals that recruit phagocytes to the dying cell, followed by “eat-me” signals that trigger ingestion (Ravichandran, 2011). Diverse candidates for find-me signals have been identified such as nucleotides, proteins, including the chemokine CX3CL1, and the lipids lysophosphatidylcholine (LPC) and sphingosine-1-phosphate (S1P) (Medina and Ravichandran, 2016); apoptotic bodies (ApoBD), which are membrane vesicles shed from apoptotic cells, have also been shown to attract phagocytes (Segundo et al., 1999; Torr et al., 2012; Eguchi et al., 2015). A well-characterized eat-me signal is the anionic phospholipid phosphatidylserine (PS), which is normally restricted to the inner leaflet of the phospholipid bilayer of the plasma membrane in healthy cells, but it becomes accessible for PS receptors expressed on phagocytes when it flips to the extracellular leaflet during apoptosis by the action of caspase-activated lipid translocases known as scramblases (Suzuki et al., 2013; Segawa and Nagata, 2015). Other complementary classes of efferocytosis signals such as “keep-out” and “good-bye” signals are also produced by apoptotic cells to prevent neutrophil recruitment and regulate gene expression in healthy neighboring cells, respectively, thereby preserving the non-phlogistic nature of apoptotic cell clearance and promoting tissue repair (Bournazou et al., 2009; Medina et al., 2020).

Despite this mechanistic framework, the precise factors that coordinate efferocytosis at the molecular level *in vivo* have not been clearly delineated. Nucleotides are the only find-me signals that have been confirmed to be required for efficient apoptotic cell clearance *in vivo* (Elliott et al., 2009; Chekeni et al., 2010). Nevertheless, since extracellular nucleotides are rapidly hydrolyzed and are known to amplify in an autocrine manner signaling induced by diverse phagocyte chemoattractants (Chen et al., 2010; Kronlage et al., 2010), they could also be acting indirectly during efferocytosis. In fact, some of these well-established find-me signals are known to play other functions unrelated to phagocyte migration. For instance, S1P has been reported to promote apoptotic cell clearance indirectly *in vivo* by activating erythropoietin signaling, which upregulates the expression of both scavenger receptors and bridging molecules by macrophages (Luo et al., 2016). Furthermore, CX3CL1 is known to upregulate MFG-E8, a PS-binding bridging molecule important for phagocytosis, in microglia and macrophages (Hanayama et al., 2004; Miksa et al., 2007; Tsai et al., 2021). Thus, the precise find-me signals that promote phagocyte migration for efferocytosis *in vivo* require further investigation and definition.

## 2 Phosphatidylserine-bound chemokines on apoptotic bodies as find-me signals in efferocytosis

Like apoptotic cells, ApoBD also expose PS on the outer membrane. ApoBD are thought to play at least three different roles in apoptotic cell clearance (Santavanond et al., 2021): 1) they provide bite-sized (1–5 µm) cell fragments that are easier for phagocytes to engulf and digest; 2) they participate in intercellular communication with phagocytes and neighboring cells; and 3) in a find-me signal-like fashion, they induce phagocyte migration by a mechanism that we showed recently involves chemokines (Pontejo and Murphy, 2021). Chemokines constitute a large family of leukocyte chemotactic proteins that signal through G protein-coupled receptors (Bachelerie et al., 2014). Given their well-established roles in leukocyte trafficking in both homeostatic and inflammatory contexts *in vivo* and the fact that cells release chemokines during apoptosis (Cullen et al., 2013), chemokines are well-suited to mediate phagocyte migration and apoptotic cell clearance *in vivo*. However, of the >40 different human chemokine ligands, only the transmembrane chemokine CX3CL1 is currently widely recognized as a find-me signal (Medina and Ravichandran, 2016). Of note, mice lacking CX3CR1, the cellular receptor for CX3CL1, displayed no defects in apoptotic cell clearance in the germinal centers (Truman et al., 2008). Therefore, the importance of CX3CL1-mediated phagocyte migration in efferocytosis remains unclear.

CXCL16, the only other transmembrane member of the chemokine ligand family, was originally identified as a PS- and oxidized LDL-binding protein and designated SR-PSOX (scavenger receptor for PS and oxidized LDL) (Shimaoka et al., 2000, 2004). Extending this concept, we have found 1) that many but not all chemokines bind directly with high affinity to PS; 2) that PS-binding chemokines bind specifically to PS exposed on the outer side of the membrane of apoptotic cells and ApoBD; and 3) that ApoBD drive phagocyte migration *ex vivo* in a chemokine-dependent manner and selectively activate chemokine receptors for PS-binding chemokines (Pontejo and Murphy, 2021). This work began with an attempt to understand how the mouse cytomegalovirus-encoded chemokine MCK2 facilitates macrophage infectivity, an activity unique to this and related cytomegalovirus chemokines (Nogalski et al., 2013; Wagner et al., 2013; Yamada et al., 2014). MCK2 did not interact with any mouse chemokine receptor, but did bind to glycosaminoglycans (GAGs) (Pontejo and Murphy, 2017), which are strongly anionic polysaccharides covalently bound to cell surface proteins on most cell types. MCK2, like most other chemokines, is strongly positively charged, which may contribute to the binding affinity. However, this interaction with GAGs did not explain MCK2-dependent macrophage infectivity (Pontejo and Murphy, 2019). We therefore initiated a search for other macrophage infectivity determinants specific for the viral chemokine MCK2 and considered phospholipid components of the plasma membrane. We broadened the search to include commercially available recombinant human chemokines, most of which are also basic proteins, and conducted a biochemical screen to an array of purified phospholipids in which 6 out of 10 human chemokines tested could bind (Pontejo and Murphy, 2021). This was confirmed by binding experiments with PS-containing liposomes and 21 different human chemokines in both ELISA-based assays and in real time by biolayer interferometry (Pontejo and Murphy, 2021).

The findings of PS binding by chemokines and chemokine-dependent phagocyte recruitment by ApoBD support a potentially important role of chemokines in efferocytosis, provide a molecular mechanism for the find-me signal activity of ApoBD, and show that PS may multitask in apoptotic cell clearance as both an eat-me signal and as an adaptor for a bona fide chemotactic factor acting as a find-me signal. In particular, we favor a model in which PS-bound chemokines on ApoBD function as haptotactic phagocyte recruitment factors.

## 3 A “breadcrumb” model for apoptotic cell clearance: ApoBD-associated chemokine gradients

Many biological processes, including immune responses, metastasis and development, rely on the efficiency conferred by directed gradients of chemoattractants to coordinate cell migration and cell-cell interactions (Schneider and Haugh, 2006; Tweedy and Insall, 2020). However, how find-me signals establish gradients to direct and orient phagocyte migration is not well defined. In contrast, chemokine action *in vivo* has been shown to rely on gradients that are formed by binding to GAGs on the surface of barrier cells, such as vascular endothelial cells (Monneau et al., 2016). Chemokines bound to GAGs are thought to be protected from protease degradation and shear forces in blood vessels that might prevent gradient establishment. Recent advances in the structural biology of chemokine ligand-receptor interactions have indicated that for most chemokines, GAG-binding is not compatible with simultaneous binding to cognate cellular receptors (Proudfoot et al., 2017). Instead, GAG-binding is critical to retain, localize and concentrate chemokines on endothelial surfaces for leukocyte transendothelial migration, but chemokines are thought to detach from GAGs before activating their G protein-coupled receptors (Graham et al., 2019). However, we found that apoptotic cells downregulate GAG expression while upregulating PS externalization and that PS-binding chemokines bind to apoptotic cells enzymatically stripped of GAGs but not when these cells were preincubated with the PS-binding protein annexin V (Pontejo and Murphy, 2021). Moreover, unlike GAG-binding, PS-binding by chemokines is compatible with simultaneous activation of cognate chemokine receptors and cell migration (Pontejo and Murphy, 2021). Thus, PS and not GAGs may be the preferred substrate for the formation of intratissue chemokine gradients in the context of efferocytosis.

**FIGURE 1:**
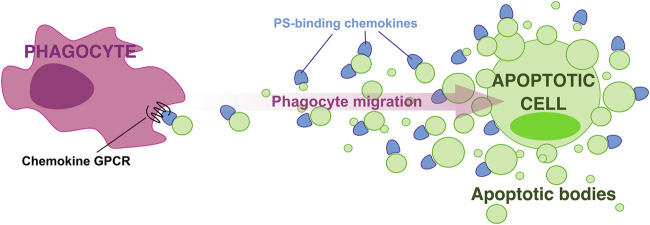
A “breadcrumb” model for directed phagocyte migration during efferocytosis. A gradient of apoptotic bodies presenting PS-bound chemokines on their surface direct phagocyte migration toward apoptotic cells.

Chemokines bind PS preferentially over other more highly anionic phospholipids (Pontejo and Murphy, 2021), which indicates that PS-chemokine binding is not simply determined by charge-charge interactions. Furthermore, we found that GAG-binding does not always correlate with PS-binding by chemokines. For instance, CCL5, a chemokine that binds GAGs with very high affinity, does not interact with PS (Pontejo and Murphy, 2021). These observations support the existence of significant structural and functional differences between GAG- and PS-binding by chemokines, and position PS as the ideal surface anionic scaffold to build chemokine gradients associated with PS-positive membranes such as those of ApoBD, which like a breadcrumb trail, may mark the path for phagocytes to locate and reach apoptotic cells. In this hypothetical “breadcrumb” model (Figure 1), chemokine binding to PS on the surface of ApoBD, with more and bigger ApoBD being retained in the proximity of the apoptotic cell while a few small chemokine-presenting vesicles manage to reach distant phagocytes, may favor the formation of steep haptotactic gradients to efficiently attract and guide phagocytes toward apoptotic cells. This novel paradigm for phagocyte migration during efferocytosis has the advantages of involving an haptotactic gradient and the chemokine signaling network whose cellular and ligand-receptor specificities are well-characterized (Bachelerie et al., 2014).

## 4 Conclusion

All three core components of the breadcrumb model, PS, ApoBD, and chemokines, have been separately proposed to play important roles in apoptotic cell clearance. However, this model implies a cooperative effect of these three elements on phagocyte migration during efferocytosis that will be challenging to demonstrate *in vivo*. Future experiments to test this model *in vivo* must distinguish between the anionic scaffold and eat-me activities of PS, the different roles attributed to ApoBD as well as potential contributions of soluble, GAG-bound, and PS-bound chemokines. Analysis of transgenic mice expressing PS-binding-defective chemokine mutants is one potential approach.

This is the first evidence that PS can regulate the bioactivity of a family of soluble cytokines by direct interaction. The discovery that ApoBD can recruit phagocytes in a PS-bound chemokine-dependent manner has broad biologic implications and supports a new mechanism for chemokine delivery by other PS-positive extracellular vesicles that mediate intercellular communications in other relevant biological contexts, ranging from cancer to infectious diseases.
